# Exploring the effects of fermented Chinese herbal medicine on growth, cecal microbiota, metabolism, and muscle flavor-related compounds in fattening pigs

**DOI:** 10.3389/fmicb.2026.1781152

**Published:** 2026-03-25

**Authors:** Xuan Luo, Jun Chen, Guofang Wu, Youli Yao, Peilin Ye, Shiyan Zhang, Jianjun Zhao, Rong Li, Yong Shi, Yongfu Liu, Huashan Mo, Lei Wang

**Affiliations:** 1 Key Laboratory of Animal Genetics and Breeding on Tibetan Plateau, Qinghai University, Xining, China; 2 Plateau Livestock Genetic Resources Protection and Innovative Utilization Key Laboratory of Qinghai Province, Qinghai University, Xining, China; 3 Life Sciences Research Center, The First Affiliated Hospital of Henan Medical University, Weihui, China; 4 Livestock and Veterinary Station, Haidong, China; 5 Bamei Pig Technical Service Center, Huzhu, China; 6 Livestock and Veterinary Station, Minhe County, China

**Keywords:** fattening pigs, fermented Chinese herbal medicine, growth performance, intestinal microbiota, meat flavor, metabolomics

## Abstract

Fermented Chinese herbal medicines (FCHMs) are considered a promising natural feed additive in livestock production. While their potential benefits for growth and health are recognized, the mechanisms underlying their effects on meat quality traits such as flavor, and the mediating role of the gut environment, remain insufficiently explored. This study aimed to investigate the multifaceted effects of FCHMs on finishing pigs, with a focus on potential links between growth, cecal microbiota, metabolism, and muscle composition. A compound FCHM was prepared by fermenting 10 traditional herbs using selected lactic acid bacteria and yeast. Eighty Duroc × Landrace × Yorkshire pigs (30.0 ± 1.0 kg) were randomly assigned to a control group (CK, basal diet) or groups supplemented with 0.4% (GL), 0.6% (GM), or 0.8% (GH) FCHM. After a 110-day trial, FCHM supplementation was associated with improved growth performance. Pigs receiving FCHM showed significantly higher final body weight and average daily gain compared to the CK group, while the high-dosage group (GH) exhibited a reduced feed-to-gain ratio. Analysis of a subset of animals revealed that FCHM appeared to reshape the cecal microbiota structure, notably decreasing the relative abundance of *Alloprevotella*, *Muribaculaceae*, and *Prevotella*, while promoting genera such as *Marvinbryantia*, *Veillonella*, and *Tyzzerella*. Concomitant shifts in cecal metabolite profiles were observed, including altered levels of compounds like cytosine, hypoxanthine, L-malic acid, and uric acid. Furthermore, analysis of muscle tissue indicated that FCHM supplementation coincided with changes in the profile of fatty acids and volatile organic compounds, which are relevant to flavor. Preliminary integrative analysis suggested potential associations between specific microbial shifts, metabolite changes, and alterations in muscle flavor-related compounds. This exploratory study indicates that dietary FCHM supplementation can enhance the growth performance of finishing pigs and is associated with concurrent modifications in the cecal microbial ecosystem, metabolic profile, and muscle composition. The observed multi-level changes suggest potential associations between gut microbial alterations and muscle characteristics that may contribute to the effects of FCHMs. These findings provide a foundational framework for understanding the complex role of fermented herbs in animal nutrition and warrant further investigation to validate the proposed mechanisms.

## 1 Introduction

Chinese herbal additives are characterized by their natural composition, multifunctional properties, non-toxicity, and absence of drug resistance (Yeh et al., 2011). These compounds not only enhance animal productivity, but also significantly improve the quality of animal-derived products (Lei et al., 2018). In addition to these benefits, Chinese herbs serve as exquisite seasonings for cooking meat, imparting distinctive flavors and aromas that enrich the overall dining experience. By utilizing these herbal additives as cooking spices, a balance between flavor and health benefits can be achieved, ensuring that dishes are both delicious and nutritious (Zhang et al., 2022; Yang et al., 2021). The combination of 10 herbs used in this study (tangerine peel, fennel, star anise, cinnamon, wolfberry, cloves, nutmeg, ginger, hawthorn, and garlic) was selected with a dual purpose. First, the combination is inspired by traditional culinary recipes commonly used for braising and stewing meats, aiming to utilize the natural flavor compounds of these herbs. Second, some of the individual herbs within this combination, such as hawthorn and wolfberry, are documented in traditional veterinary practice for their roles in promoting digestion and enhancing appetite. Through fermentation with probiotics, we aimed to further improve the bioavailability of their bioactive components and potential health benefits. Previous studies have shown that supplementing the diet of Songliao black pigs with fermented herbal medicines, such as ginseng stems and leaves, Polygonatum odoratum, and licorice, during the finishing phase reduces backfat thickness, increases curing yield and oleic acid content, thereby improving pork quality and nutritional value (Liu et al., 2023). Additionally, adding cordyceps fungus liquid fermented herbal culture medium to the diet can significantly improve the growth performance and carcass quality of growing pigs (Dai et al., 2011). Fermented herbal preparations have been demonstrated to boost immune function and improve the growth performance of pigs (Liu et al., 2024). Moreover, Chinese herbal additives have the potential to improve meat quality by reducing muscle fiber diameter and increasing intermuscular fat content (Tan et al., 2002). Recent findings suggest that herbal additives can also enhance meat flavor by altering the amino acid content within muscle tissue. However, research on the combined effects of probiotic fermentation and herbal medicines on pork quality remains limited (Wu et al., 2009).

The gut microbiota, often referred to as the “second genome” or the “forgotten functional organ,” plays a pivotal crucial in animal health, growth, development and metabolic functions (O'Hara and Shanahan, 2006; Santo et al., 2021). Maintaining the balance of the gut microbiota is essential for effective nutrient absorption and overall host health (Bischoff, 2011). Research indicates that gut-derived flavor compounds, originating from the microbial metabolic byproducts, can transfer to muscle tissue (Wu et al., 2022). In pigs, dietary fiber is primarily fermented in the large intestine, and the resulting volatile fatty acids are absorbed by the cecum and colon (Gargallo and Zimmerman, 1980). Variations in nutrient supply can lead to differences in the microbial community structure. Fermented diets have been shown to enhance gastrointestinal health and prevent clinical diseases by reducing gastric pH and suppressing pathogenic bacteria (Boesen et al., 2004). Furthermore, probiotics offer multiple benefits for animal behavior, including feed intake optimization, improvement of rumen microbiota structure and function, and enhanced weight gain in livestock (Schofield et al., 2018). Certain probiotics may also facilitate fat utilization by oxidizing fat into smaller molecular entities, thereby enhancing meat flavor and quality. Additionally, flavor compounds from microbial metabolites are absorbed by livestock and poultry, accumulating in muscle tissues and influencing lipid metabolism (Sun et al., 2018).

Against the backdrop of evolving consumer preferences and a growing emphasis on dietary health, understanding the factors that influence meat quality and flavor has gained considerable research interest. This study was therefore designed to explore the multifaceted effects of supplementing the diet of finishing pigs with probiotic-fermented Chinese herbal medicine (FCHM). We aimed to systematically evaluate its influence on growth performance, carcass traits, and meat composition, with a particular focus on investigating the potential links between these outcomes and concurrent changes in the cecal microbiota and metabolome. By analyzing these interrelationships, this work seeks to provide preliminary mechanistic insights into how FCHM may modulate host physiology and muscle characteristics, thereby contributing to the scientific basis for its potential application in animal production. Specifically, we hypothesize that FCHM may exert its beneficial effects through a series of associated physiological changes: (i) by remodeling the cecal microbial community structure; (ii) by modulating associated microbial metabolic functions, particularly in pathways such as purine metabolism and the tricarboxylic acid cycle; and (iii) that these gut-level alterations coincide with observable improvements in muscle composition, including the fatty acid profile and volatile flavor compound spectrum.

## 2 Materials and methods

### 2.1 Experimental materials and preparation

Standard herbal compounds (Tangerine peel, fennel, star anise, cinnamon, wolfberry, cloves, nutmeg, ginger, hawthorn and garlic powder) were purchased from Hubei Fushengtang Traditional Chinese Medicine Co., Ltd., and the content of Chinese herbal medicine is shown in Supplementary Table S1. Three strains of lactic acid bacteria (*Lactobacillus rhamnosus*, *Pediococcus acidilactici*, *Lactobacillus casei*), and two strains of yeast (*Saccharomyces cerevisiae*, *Pichia* yeast) were meticulously screened and purified by the Plateau Livestock Genetic Resources Protection and Innovative Utilization Key Laboratory of Qinghai Province, as previously reported (Supplementary Table S2) (Wan et al., 2022). After procurement, the herbal medicines were dried, subjected to gradient dilution, and the total viable microbial count was determined using the plate spread method.

The viable bacteria count for the three lactic acid bacteria strains was determined to be ≥6 × 10^11^ CFU/mL, while the two yeast strains maintained a viable bacteria count of ≥1 × 10^8^ CFU/mL. A seeding rate of 10% was applied, with the five strains evenly distributed in a 1:1:1:1:1 ratio. The mixture was prepared using a water-to-material ratio of 0.60:1 and fermented in a sealed fermenter at 25 °C for 5 days (Cao, 2023). The nutritional components, bioactive substances, and toxin levels in the feed before and after fermentation are shown in Supplementary Table S3.

### 2.2 Animals and modeling

#### 2.2.1 Animal ethics

The experiment utilized Duroc × Landrace ×Yorkshire pigs with an average weight of 30.00 ± 1.00 kg at Qinghai Yufu Animal Husbandry Development Co., Ltd. Subsequent experiments were conducted at Key Laboratory of Animal Genetics and Breeding on Tibetan Plateau, and the Plateau Livestock Genetic Resources Protection and Innovative Utilization Key Laboratory of Qinghai Province. The research strictly adhered to the “Animal Care and Use Guidelines” established by Qinghai University’s Institutional Animal Care and Use Committee (IACUC). Furthermore, the protocol employed in the experiments was approved by the Animal Ethics Committee of Qinghai University (Approval number: NQH2019102).

#### 2.2.2 Experimental design

Eighty pigs were managed under uniform conditions and randomly assigned to four groups, each with four replicates of five pigs. Control group (CK): basal diet, low-concentration group (GL): basal diet + 0.4% fermented herbal medicine, moderate-concentration group (GM): basal diet + 0.6% fermented herbal medicine, high-concentration group (GH): basal diet + 0.8% fermented herbal medicine. The basal diet was prepared in accordance with NRC standards (2012), with details of its composition and nutritional values provided in Supplementary Table S4. The experiment included a 7-day preliminary adaptation period, followed by a 110-day trial. During the experimental period, a total of 4 pigs were removed based on veterinary advice due to sporadic health issues unrelated to the dietary treatments, primarily poor appetite and locomotion. Specifically, 2 pigs were removed from the control group (CK), and 1 pig each was removed from the low-concentration (GL) and moderate-concentration (GM) groups. The health issues leading to removal were confirmed to be independent of the intake of the fermented herbal medicine additive. Consequently, the final number of pigs that completed the trial and were included in the growth performance analysis was: CK = 18, GL = 19, GM = 19, GH = 20 (Table 1). From the trial’s inception, daily feed intake and remaining food quantities were tracked to calculate the average daily feed intake (ADFI). The pigs’ fasting weights at both the beginning and end of the trial were recorded to compute the average daily gain (ADG) and feed gain ratio (F/G). Pigs were randomly selected from each group and slaughtered following a 24-h fasting period. Carcass weight, backfat thickness, loin-eye area, drip loss, cook yield, shear force, pH45-min value and muscle color were measured. Cecal content specimens were collected for 16S rRNA gene sequencing and metabolome analysis.

**Table 1:** The effect of adding different levels of fermented Chinese herbal medicine on the growth performance of fattening pig.

Items	CK^2^	GL	GM	GH
Numbers	18	19	19	20
Initial weight/kg^1^	29.86 ± 0.42	30.03 ± 0.36	29.92 ± 0.49	29.77 ± 0.41
Final weight/kg	120.10 ± 6.52^b^	139.80 ± 8.44^a^	134.90 ± 8.12^a^	137.60 ± 5.54^a^
Average daily feed intake/kg	2.22 ± 0.79^a^	2.06 ± 0.79^ab^	2.07 ± 0.77^ab^	1.97 ± 0.73^b^
Average daily gain/g	805.71 ± 57.62^b^	980.09 ± 74.94^a^	937.32 ± 73.06^a^	962.77 ± 49.62^a^
Feed conversion ratio	2.76 ± 0.20:1^a^	2.11 ± 0.16:1^b^	2.22 ± 0.17:1^bc^	2.05 ± 0.11:1^c^

1 Different lowercase letters in the same row indicate significant differences (*p* < 0.05). Data represent the mean ± standard deviation of the number of surviving pigs in each group. The final number of pigs per group: CK = 18, GL = 19, GM = 19, GH = 20.

2 CK, control group; GL, low-concentration group; GM, moderate-concentration group; GH, high-concentration group.

#### 2.2.3 Slaughter procedure and sample collection

At the end of the 110-day feeding trial, pigs (one pig per replicate, *n* = 3 per treatment group for carcass and meat quality analysis) with body weights close to the group average were selected for slaughter. After a 24-h fasting period with free access to water, pigs were humanely stunned using electrical stunning followed by exsanguination, according to standard commercial protocols. Carcasses were scalded, dehaired, and eviscerated. Immediately after evisceration, the following carcass measurements were taken: Carcass weight was recorded. Backfat thickness was measured at the 10th–11th rib interface using a digital caliper. The cross-sectional area of the longissimus dorsi (LD) muscle was traced onto acetate paper, and the loin eye area was calculated based on the measured length and width of the LD muscle cross-section. From the left side of each carcass, a section of the LD muscle spanning the 10th to the 12th ribs was excised. This sample was immediately used for meat quality measurements and then subdivided. One portion was snap-frozen in liquid nitrogen and stored at −80 °C for subsequent analysis of fatty acids and volatile compounds. Another portion was used for immediate meat quality assessment as described below. Additionally, cecal content was aseptically collected from the same slaughtered animals (with a subset selected for omics analyses, see sections 2.4 and 2.5), immediately snap-frozen in liquid nitrogen, and stored at −80 °C for microbiota and metabolomics analysis.

#### 2.2.4 Measurement of carcass traits and meat quality

Meat quality measurements were conducted on the fresh LD muscle samples. pH at 45 min post-mortem (pH 45 min) was measured using a portable pH meterby inserting the probe into the LD muscle. Meat color (L, a, b*) was assessed on a freshly cut surface after a 30-min blooming period at 4 °C using a chroma meter. Drip loss was determined using the bag suspension method. A core of LD muscle (approximately 5 cm × 3 cm × 2 cm) was weighed (W1), suspended in a sealed plastic bag at 4 °C for 24 h, then removed, blotted dry, and re-weighed (W2). Drip loss percentage was calculated as [(W1 − W2)/W1] × 100%. Cooking loss was assessed by weighing a ~100 g sample (W3), vacuum-sealing it in a plastic bag, cooking it in a water bath at 80 °C until the core temperature reached 70 °C, cooling it in ice water, drying the surface, and re-weighing (W4). Cooking loss percentage was calculated as [(W3 − W4)/W3] × 100%. Shear force was measured as an indicator of tenderness. The cooked sample used for cooking loss was used. It was cooled to room temperature, and then cylindrical cores (1 cm diameter) were taken parallel to the muscle fiber orientation. Each core was sheared perpendicular to the fiber direction using a texture analyzer equipped with a Warner-Bratzler shear blade. The peak force (kg·F or N) required to shear the sample was recorded, and the average of at least three cores per sample was used for statistical analysis.

### 2.3 Targeted metabolomics profiling

#### 2.3.1 Fatty acid composition analysis in muscle

For this analysis, samples were collected from all pigs within each treatment group at slaughter. Accurately weigh 1 g of homogenized longissimus dorsi muscle and add 5 mL of the extraction solution. Mix thoroughly by high-speed shaking and place the mixture in a 50 °C water bath for ultrasonic extraction for 90 min. Centrifuge the sample at 4,000 rpm, transfer the supernatant, repeat the extraction once, and combine the extracts. Add 2 mL of saturated sodium chloride solution, shake and mix for 30 s, centrifuge at 4,000 rpm for 10 min, and transfer the lower chloroform layer. Add 0.5 g of anhydrous sodium sulfate to dry, shake and mix for another 30 s, followed by another centrifugation at 4,000 rpm for 10 min. Transfer the supernatant and dry the chloroform under nitrogen at 50 °C to obtain the fat. Add 5 mL of hexane and 3 mL of potassium hydroxide methanol, shake well, and perform methylation in a 60 °C oven for 30 min. Centrifuge at 4,000 rpm for 10 min, take 1 mL of the supernatant, and filter it using a 0.22 μm membrane for analysis. The primary instrument used is the GC-7890, with a DB-FFAP (60 × 0.25 mm) chromatography column. An FID detector with a 1 μL injection volume was utilized. The post-detector temperature was maintained at 280 °C, the injection port at 250 °C, and the flow rate at 1 mL/min. The column temperature was set at 180 °C with a gas ratio of H₂: Air: N₂ = 40:400:40 mL/min in split mode at 50:1.

#### 2.3.2 Volatile flavor compounds analysis in meat

Accurately weigh 3 g of muscle and cut it into small cubes to be placed in a headspace vial. Incubate the vial in a 90 °C oil bath for 40 min. Insert the solid-phase microextraction (SPME) needle into the vial, extending the fiber by deploying the handle rod, which allows the fiber to reach equilibrium during the absorption process. Retract the fiber by pulling back the rod and then remove the needle, inserting it into the GC/MS injection port. Extend the fiber by pushing the handle rod to prepare the sample for thermal desorption analysis. The GC conditions are as follows: the temperature program starts at 35 °C for 2 min, then increases to 230 °C at a rate of 5 °C/min, and is held for another 5 min. The vaporization is performed at 250 °C with a helium carrier gas flow rate of 1.0 mL/min and involves no split. The MS operation uses an electron impact (EI) ion source with an electron energy of 70 eV. Additional conditions include the transfer line, ion source, and interface temperatures set at 220 °C, 200 °C and 250 °C, respectively. The procedure concludes with a mass range scan from *m*/*z* 33 to 500.

### 2.4 Quantification of cecum microbiota and analysis of bacterial diversity

#### 2.4.1 DNA extraction and sequencing

To investigate the effects on the gut microbial ecosystem, cecal content samples from a representative subset of pigs were analyzed. Specifically, three pigs per treatment group (*n* = 3/group) were selected for microbial community and subsequent metabolomic profiling. These pigs were not chosen randomly; instead, they were strictly selected based on having final body weights closest to the group average, thereby minimizing inter-individual variation and ensuring that the microbial and metabolic data captured were representative of the typical response within each treatment group. Total genomic DNA of the microbial community is extracted following the instructions of the E.Z.N.A.® Soil DNA Kit (Omega Bio-Tek, Norcross, GA, U.S.). The quality of the extracted DNA is verified using 1% agarose gel electrophoresis, and the concentration and purity are assessed with a NanoDrop 2000 spectrophotometer (Thermo Scientific, U.S.). The V3-V4 variable region of the 16S rRNA gene is amplified via PCR using upstream primer 338F (5′-ACTCCTACGGGAGGCAGCAG-3′) and downstream primer 806R (5′-GACTACHVGGGTWTCTAAT-3′), each containing a barcode sequence. The PCR reaction conditions are as follows: DNA (10 ng) is used as a template. Samples are mixed with 5 × TransStart buffer (2 μL), 2.5 mM dNTPs (2 μL), upstream primer (0.8 μL), downstream primer (0.8 μL), and TransStart FastPfu DNA Polymerase (0.4 μL). The mixture is heated to 95 °C for 3 min, followed by 27 cycles of 95 °C for 30 s, 50 °C for 30 s, and 72 °C for 30 s.

#### 2.4.2 Sequencing information analysis

Raw sequencing reads were processed using a standardized pipeline within QIIME2 (version 2020.2). According to the fastp software, the double ended raw sequencing sequences were quality controlled (Chen et al., 2018). The sequences were then spliced using the FLASH software (Magoc and Salzberg, 2011) and denoised using the DADA2 plugin (Callahan et al., 2016) within the Qiime2 process (Bolyen et al., 2019) framework. After quality control and splicing, the sequence succeeding DADA2 denoising is typically known as Amplification Sequence Variants (ASVs). Annotations pertaining to chloroplast and mitochondrial sequences were eliminated from all samples to minimize the impact of sequencing depth on ensuing Alpha and Beta diversity data analysis. After random flattening of all sample sequences, the average sequence coverage reached approximately 99.09%. The ASVs underwent species taxonomy analysis using the Naive Bayes classifier within the Qiime2, based on the Sliva 16S rRNA gene database.

### 2.5 LC–MS/MS-based metabolomic analysis of cecal contents

Cecal content samples (from the same subset of animals described in section 2.4.1, selected using the same body weight-based criterion, *n* = 3/group) were subjected to untargeted metabolomic profiling, using an LC–MS platform (ThermoFisher, Waltham, MA, USA). Approximately 100 mg of sample was homogenized in liquid nitrogen and extracted with 80% pre-cooled methanol containing 0.1% formic acid. After a 5-min incubation on ice, the samples were centrifuged at 15,000 rpm for 5 min at 4 °C. The supernatant was diluted with LC–MS grade water to lower the methanol concentration to 60%. The samples were then transferred to a clean microcentrifuge tube with a 0.22 μm filter and centrifuged for 10 min at 4 °C. The filtered solution was subsequently used for LC–MS/MS analysis.

### 2.6 Statistical analysis

All experimental data were processed and analyzed by GraphPad Prism software (8.0.2, ©1992–2019, GraphPad Software Inc., San Diego, CA, USA) and SAS software (©2001, Indigo Rose, Corporation). Quantitative data are presented as means ± standard deviation (SD). Prior to statistical analysis, the normality of data distribution for all continuous variables was assessed using the Shapiro–Wilk test, and homogeneity of variances across groups was evaluated using Levene’s test. When both assumptions were met (*p* > 0.05), one-way analysis of variance (ANOVA) followed by Duncan’s multiple range test was employed to assess the main effects of treatments. For data that did not satisfy the assumptions of normality or homogeneity of variance, non-parametric Kruskal-Wallis tests followed by Dunn’s post-hoc tests were applied. A *p*-value of less than 0.05 was considered to indicate statistical significance. Each measurement was performed with at least three technical replicates.

## 3 Results

The trial began with 80 pigs (20 per group). During the 110-day experimental period, a total of 4 pigs were removed based on veterinary advice due to sporadic health issues unrelated to the dietary treatments. The clinical signs leading to removal were primarily manifested as poor appetite and reduced locomotion, which are common sporadic occurrences in commercial pig production. Specifically, 2 pigs were removed from the control group (CK), and 1 pig each was removed from the low-concentration (GL) and moderate-concentration (GM) groups. The health issues leading to removal were confirmed by veterinary assessment to be independent of the intake of the fermented herbal medicine additive. Notably, no removals occurred in the high-dose FCHM group (GH), further supporting the safety profile of the supplement. Consequently, the final number of pigs that completed the trial and were included in the growth performance analysis was: CK = 18, GL = 19, GM = 19, GH = 20. All slaughter and sampling procedures were subsequently performed on healthy, representative individuals from these final groups.

### 3.1 Growth performance of fattening pigs

At the conclusion of the experimental period, data on the growth performance of fattening pigs were compiled. As shown in Table 1, pigs in the experimental group (GL, GM, GH), which received fermented Chinese herbal medicine supplementation, exhibited significantly higher final weight (FW) and average daily gain (ADG) compared to the CK (*p* < 0.05). Furthermore, there was a significant reduction in both average daily feed intake (ADFI) and the feed-to-gain ratio (F/G) in the GH (*p* < 0.05).

### 3.2 Carcass performance and meat quality of fattening pigs

Dietary inclusion of fermented Chinese herbal medicine in the basic diet significantly increased carcass weight in all experimental groups compared to the CK (*p* < 0.05), as shown in Table 2, the GL achieving the highest carcass weight, and the GM exhibited a significantly smaller ribeye area compared to the CK (*p* < 0.05). Groups supplemented with fermented Chinese herbal medicine also demonstrated a significantly higher cooked meat rate than the CK. Additionally, there was a decreasing trend in backfat thickness and muscle shear force. No significant differences were observed among the groups in terms of slaughter rate, muscle pH_45min_, meat color or water-holding capacity (*p* > 0.05).

**Table 2:** The effect of fermented Chinese herbal medicine on carcass quality and meat quality of growing and fattening pigs.

Items		CK^2^	GL	GM	GH
Carcass weight (kg)^1^		81.47 ± 1.63^c^	102.40 ± 5.41^a^	92.80 ± 2.43^b^	93.27 ± 3.93^b^
Dressing percentage (%)		67.85 ± 4.90	73.44 ± 5.13	69.28 ± 4.06	67.73 ± 0.53
Backfat thickness (cm)		3.56 ± 0.58	3.33 ± 0.51	2.74 ± 0.15	2.77 ± 0 0.50
ribeye area (cm^2^)		4.09 ± 0.34^a^	3.71 ± 0.15^a^	3.13 ± 0.07^b^	3.92 ± 0.39^a^
Shear force (kg·F)		60.68 ± 7.88	55.98 ± 7.30	58.21 ± 4.88	47.94 ± 0.71
pH_45min_		6.26 ± 0.16	6.34 ± 0.07	6.39 ± 0.06	6.43 ± 0.08
Drip loss (%)		5.41 ± 2.47	7.72 ± 0.95	6.10 ± 2.50	7.26 ± 3.53
Cooked meat yield (%)		62.27 ± 3.26^b^	68.05 ± 0.24^a^	70.52 ± 2.37^a^	73.11 ± 4.38^a^
Meat color	L*	47.43 ± 1.01	43.46 ± 3.00	45.84 ± 1.98	44.90 ± 1.19
	A*	5.97 ± 0.58	5.73 ± 1.33	6.68 ± 2.85	5.45 ± 1.10
	B*	4.55 ± 0.64	3.86 ± 2.18	4.75 ± 1.94	3.72 ± 0.70

1 Different lowercase letters in the same row indicate significant differences (*p* < 0.05). Data represent the mean ± standard deviation of the number of surviving pigs in each group. The final number of pigs per group: CK = 18, GL = 19, GM = 19, GH = 20.

2 CK, control group; GL, low-concentration group; GM, moderate-concentration group; GH, high-concentration group.

### 3.3 Determination of diverse fatty acids concentration in the longissimus dorsi muscle tissue

Among the nine saturated fatty acids, palmitic acid and stearic acid are predominant, while oleic acid and linoleic acid are the main unsaturated fatty acids (Table 3). Compared to the CK, the concentrations of C12:0, C22:0, C18:3, C20:3, C20:4, C20:5 and C22:6 were significantly lower in all experimental groups (*p* < 0.05). The levels of C16:1 and C18:1 were significantly higher than in the CK (*p* < 0.05). Notably, the GL exhibited a marked reduction in C17:0 and C18:2 levels (*p* < 0.05). Furthermore, both polyunsaturated fatty acids (PUFA) and the PUFA to saturated fatty acid (SFA) ratio were significantly reduced in the experimental groups (*p* < 0.05).

**Table 3:** Effects of different levels of fermented Chinese herbal medicine on fatty acid content of fattening pigs.

Items	CK^2^	GL	GM	GH
C8:0	0.12 ± 0.02	0.11 ± 0.01	0.10 ± 0.02	0.11 ± 0.01
C12:0^1^	0.15 ± 0.02^a^	0.09 ± 0.02^b^	0.09 ± 0.01^b^	0.09 ± 0.01^b^
C14:0	1.33 ± 0.11	1.46 ± 0.09	1.48 ± 0.07	1.47 ± 0.05
C15:0	0.07 ± 0.02^a^	0.05 ± 0.01^ab^	0.07 ± 0.01^a^	0.05 ± 0.00^ab^
C16:0	25.33 ± 0.10	25.56 ± 1.23	25.46 ± 1.21	27.24 ± 0.58
C17:0	0.20 ± 0.01^a^	0.15 ± 0.01^b^	0.21 ± 0.01^a^	0.20 ± 0.01^a^
C18:0	15.15 ± 0.28	15.04 ± 0.79	15.29 ± 1.48	16.01 ± 0.74
C20:0	0.25 ± 0.01	0.26 ± 0.02	0.28 ± 0.01	0.25 ± 0.02
C22:0	0.22 ± 0.07^a^	0.08 ± 0.01^b^	0.1 ± 0.01^b^	0.07 ± 0.01^b^
C16:1	2.40 ± 0.10^b^	3.14 ± 0.07^a^	3.10 ± 0.28^a^	3.34 ± 0.42^a^
C18:1	41.23 ± 0.60^b^	45.69 ± 2.21^a^	45.64 ± 1.09^a^	43.26 ± 1.90^a^
C18:1 T	0.15 ± 0.02^bc^	0.19 ± 0.03^a^	0.18 ± 0.01^ab^	0.13 ± 0.01^c^
C20:1	0.76 ± 0.06	0.88 ± 0.07	0.95 ± 0.09	0.81 ± 0.11
C22:1	0.22 ± 0.09	0.20 ± 0.03	0.23 ± 0.02	0.17 ± 0.07
C18:2	7.67 ± 0.11^a^	5.96 ± 0.44^b^	6.36 ± 0.89^ab^	6.53 ± 0.93^ab^
C18:3 T	0.16 ± 0.01^a^	0.05 ± 0.01^b^	0.06 ± 0.01^b^	0.05 ± 0.01^b^
C18:3	0.20 ± 0.03	0.15 ± 0.02	0.17 ± 0.01	0.20 ± 0.04
C20:2	0.31 ± 0.09	0.23 ± 0.01	0.28 ± 0.05	0.25 ± 0.01
C20:3	0.27 ± 0.05^a^	0.16 ± 0.02^b^	0.18 ± 0.02^b^	0.15 ± 0.04^b^
C20:4	1.70 ± 0.47^a^	0.82 ± 0.05^b^	1.03 ± 0.2^b^	0.96 ± 0.07^b^
C20:5	0.10 ± 0.03^a^	0.05 ± 0.01^b^	0.06 ± 0.02^b^	0.04 ± 0.01^b^
C22:6	0.13 ± 0.06^a^	0.05 ± 0.01^b^	0.06 ± 0.02^b^	0.07 ± 0.01^b^
∑SFA^3^	42.31 ± 1.01	42.77 ± 1.79	43.05 ± 1.53	44.72 ± 2.05
∑MUFA^4^	46.48 ± 2.97	50.09 ± 2.24	49.81 ± 1.10	47.71 ± 2.02
∑PUFA^5^	10.79 ± 0.94^a^	7.48 ± 0.43^b^	8.19 ± 1.02^b^	8.25 ± 1.03^b^
PUFA/SFA	0.26 ± 0.24^a^	0.17 ± 0.01^b^	0.19 ± 0.03^b^	0.19 ± 0.04^b^

1 Different lowercase letters in the same row indicate significant differences (*p* < 0.05). Data represent the mean ± standard deviation of the number of surviving pigs in each group. The final number of pigs per group: CK = 18, GL = 19, GM = 19, GH = 20.

2 CK, control group; GL, low-concentration group; GM, moderate-concentration group; GH, high-concentration group.

3 SFA = (C22:0 + C16:0 + C15:0 + C17:0 + C14:0 + C8:0 + C18:0 + C12:0 + C20:0), saturated fatty acids.

4 MUFA = (C18:1 T + C16:1 + C22:1 + C18:1 + C20:1), monounsaturated fatty acids.

5 PUFA = (C20:2 + C20:3 + C22:6 + C18:3 + C18:3 T + C18:2 + C20:4 + C20:5), polyunsaturated fatty acids.

### 3.4 Volatile flavor compounds in the longissimus dorsi muscle

Gas chromatographic analysis identified 32 volatile flavor compounds in the porcine longissimus dorsi muscle, predominantly comprising 17 aldehydes, 6 alcohols, 6 hydrocarbons, 1 ester and 2 other compounds, as shown in Table 4. Compared to the CK, the experimental groups exhibited significant decreases in hexanal, trans-2-octenal, heptanal, 1-octen-3-ol and 6-methyl-3-octyne (*p* < 0.05), while there were notable increases in tetradecanal, pentadecanal, hexadecanal, lauryl alcohol and 2,6-bis(trimethylsilyl)benzoic acid trimethylsilyl ester (*p* < 0.05). Specifically, in the GL, levels of benzaldehyde, decanal, trans-2-decenal, undecanal, tridecanal and trans-2-octenol were significantly elevated compared to the CK (*p* < 0.05), with the particularly large variation observed for trans-2-decenal likely reflecting inter-individual differences; the biological significance of this specific change warrants further verification in a larger sample size. In contrast, heptanol levels were significantly reduced in the GM (*p* < 0.05), and the GH showed a significant decrease in hexadecyl dimethyl tertiary amine (*p* < 0.05).

**Table 4:** The effect of fermented Chinese herbal medicine on volatile flavor compounds in the longest back muscle of fattening pigs.

Compound Category	Compound Name	CK^2^	GL	GM	GH
Aldehyde	Hexanal^1^	25.39 ± 1.68^a^	7.60 ± 0.67^d^	16.29 ± 0.43^c^	20.44 ± 0.02^b^
	Benzaldehyde	2.68 ± 0.12^b^	4.04 ± 0.42^a^	2.92 ± 0.70^b^	2.31 ± 0.12^b^
	Octanal aldehyde	6.12 ± 0.87	6.97 ± 0.01	5.39 ± 0.81	6.42 ± 0.02
	Trans-2-octenal	4.70 ± 0.59^a^	2.91 ± 0.30^c^	4.57 ± 0.96^ab^	3.46 ± 0.21^bc^
	Decanal	0.84 ± 0.12^b^	1.12 ± 0.05^a^	0.90 ± 0.05^b^	0.80 ± 0.02^b^
	Trans-2-decenal	1.94 ± 0.32^b^	5.58 ± 2.41^a^	–	–
	Heptanal	3.57 ± 0.56^a^	3.29 ± 0.34^a^	2.01 ± 0.14^b^	2.31 ± 0.08^b^
	Undecyl aldehyde	0.50 ± 0.14^b^	0.68 ± 0.14^a^	0.49 ± 0.03^b^	0.42 ± 0.01^b^
	Dodecanal	1.17 ± 0.26	1.21 ± 0.32	1.13 ± 0.14	0.84 ± 0.02
	Thirteen aldehydes	1.32 ± 0.42^b^	2.10 ± 0.62^a^	1.02 ± 0.04^b^	0.81 ± 0.02^b^
	Tetradecanal	1.08 ± 0.22^c^	2.38 ± 0.10^a^	1.63 ± 0.23^b^	1.47 ± 0.12^b^
	Pentadecanal	1.25 ± 0.07^c^	3.53 ± 0.97^a^	2.21 ± 0.12^b^	2.27 ± 0.22^b^
	Hexadecanal	2.74 ± 0.22^c^	2.92 ± 0.05^c^	4.96 ± 0.17^b^	5.60 ± 0.03^a^
	Pentaaldehyde	–	–	1.94 ± 0.016^a^	1.52 ± 0.06^b^
	Nonanal	–	26.40 ± 1.41^a^	15.04 ± 0.49^b^	13.57 ± 1.03^b^
	Decanal	–	–	3.74 ± 0.58^a^	1.51 ± 0.09^b^
	Undecylenic aldehyde	–	1.35 ± 0.28^b^	2.08 ± 0.33^a^	0.89 ± 0.03^c^
Alcohols	Heptanol	0.87 ± 0.10^a^	0.79 ± 0.08^ab^	0.69 ± 0.04^b^	0.78 ± 0.07^ab^
	1-octen-3-ol	7.70 ± 0.74^a^	5.83 ± 0.56^bc^	5.13 ± 0.76^c^	6.55 ± 0.09^b^
	Trans-2-octenol	0.85 ± 0.12^b^	1.09 ± 0.06^a^	0.83 ± 0.10^b^	0.81 ± 0.05^b^
	Octanol	3.82 ± 0.12^a^	–	–	0.90 ± 0.01^b^
	Laurel alcohol	0.23 ± 0.04^c^	0.28 ± 0.06^bc^	0.34 ± 0.03^b^	9.17 ± 0.02^a^
	Dimethylsilane diol	–	6.68 ± 0.93^b^	15.04 ± 0.68^a^	3.36 ± 0.01^c^
Hydrocarbons	Dodecane	1.95 ± 0.51	–	1.47 ± 0.28	1.58 ± 0.26
	Tetradecane	0.38 ± 0.18	–	–	–
	Pentadecane	–	0.39 ± 0.07^ab^	0.49 ± 0.12^a^	0.33 ± 0.11^b^
	Dodecylmethyldiethoxysilane	–	–	2.01 ± 0.15^a^	1.36 ± 0.11^b^
	4-Ethyl-3-nonen-5-yne	1.16 ± 0.13^c^	1.79 ± 0.32^ab^	2.17 ± 0.46^a^	1.60 ± 0.05^bc^
	6-methyl-3-octyne	1.77 ± 0.18^a^	1.12 ± 0.18^b^	1.21 ± 0.18^b^	1.25 ± 0.18^b^
Esters	2,6-bis (trimethylsiloxy) benzoic acid trimethylsilyl ester	1.30 ± 0.21^c^	1.50 ± 0.41^b^	3.11 ± 0.22^a^	1.46 ± 0.31^b^
Other categories	Hexadecyl dimethyl tertiary amine	2.71 ± 0.49^a^	2.64 ± 0.41^a^	2.31 ± 0.32^ab^	1.81 ± 0.06^b^
	N-pentyl furan	4.48 ± 0.47	5.06 ± 0.02	4.57 ± 0.58	4.2 ± 0.19

1 Different lowercase letters in the same row indicate significant differences (*p* < 0.05). Data represent the mean ± standard deviation of the number of surviving pigs in each group. The final number of pigs per group: CK = 18, GL = 19, GM = 19, GH = 20.

2 CK, control group; GL, low-concentration group; GM, moderate-concentration group; GH, high-concentration group.

### 3.5 Cecal microbial abundance analysis

**Figure 1:**
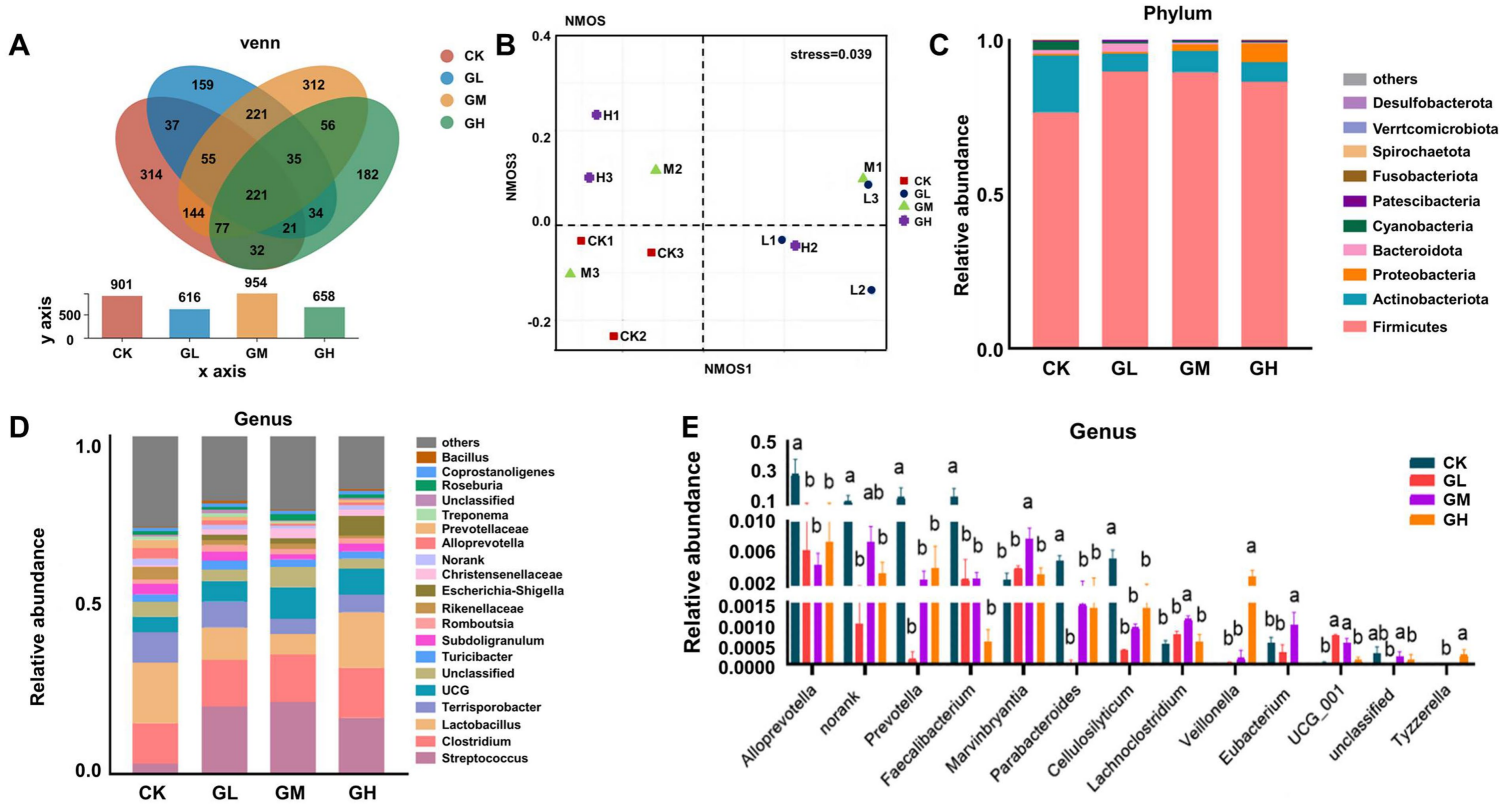
Cecal microbial abundance analysis. **(A)** Venn diagram of ASV clustering in the cecal contents. **(B)** Non-metric multidimensional scaling analysis (NMDS) of cecal microbiota. **(C)** Changes in the microbial community at a phylum level. **(D)** Changes in the microbial community at a genus level. **(E)** Significant differences in microbial communities at the genus level. GL, low-concentration group, GM, moderate-concentration, GH, high-concentration; CK, control group. Each group has three biological replicates.

ELISA The analysis of assignment statistics vector (ASV) clustering in the cecal contents identified 904 ASVs in the CK, 616 in GL, 954 in GM and 658 in GH (Figure 1A). To evaluate the similarity of microbial community structures among individuals and the potential relationship between microbiome composition and the addition of fermented herbal medicines, a non-metric multidimensional scaling analysis (NMDS) was conducted. The results show that the CK exhibits a clear separation from the GL, GM and GH. This suggests variations in the cecal microbiome of fattening pigs under different diets involving fermented herbal medicines (Figure 1B). Figure 1C illustrates the top 10 most abundant phyla, highlighting Firmicutes and Actinobacteriota as the most dominant and revealing significant differences in microbial structures between the control and experimental groups.

At the genus level, CK showed a significantly greater abundance of *Alloprevotella*, *Muribaculaceae*, *Faecalibacterium*, *Prevotella*, *Parabacteroides* and *Cellulosilyticum* compared to the GL, GM and GH (*p* < 0.05) (Figure 1D). Furthermore, in the GM, the abundance of *Marvinbryantia*, *Lachnoclostridium*, *Eubacterium* was significantly higher than in the other groups (*p* < 0.05), and in the GH, the abundance of *Veillonella* and *Tyzzerella* was significantly higher than in the other groups (*p* < 0.05).

### 3.6 Analysis of cecal metabolomic profiles

**Figure 2:**
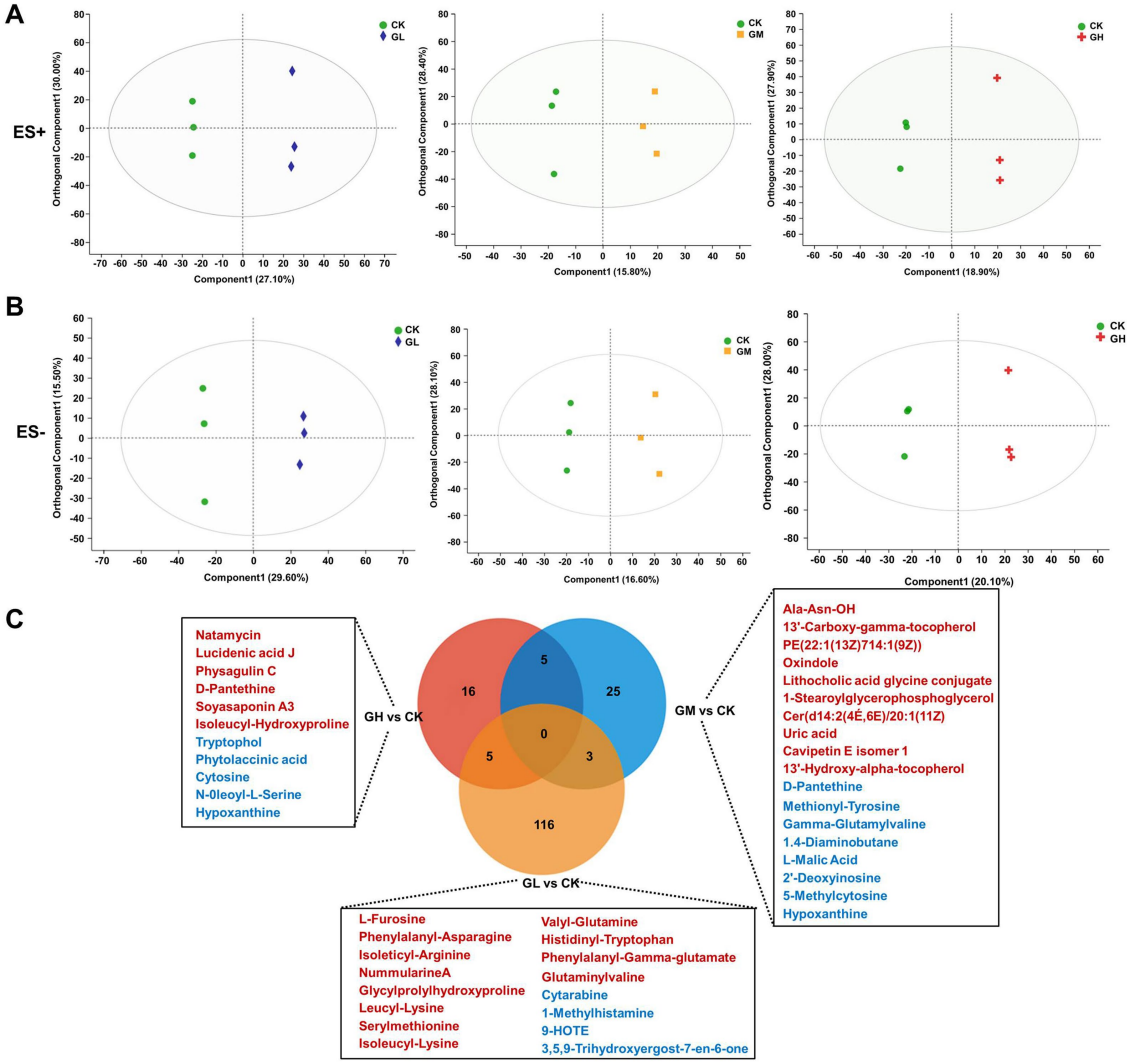
The differential metabolic characteristics of the microbiota in the cecal intestines. **(A,B)** Orthogonal partial least squares discriminant analysis (OPLS-DA) plots of negative and positive ion patterns in samples from three different dietary groups and control group. Green dot, CK group data. Purple dot, GL group data. Yellow dot, GM group data. Red dot, L group data. **(C)** The Venn diagram displays the differential metabolites associated with the KEGG metabolic pathway in the intestinal contents when CK with GL, GM, and GH conditions. Red and blue represent the upregulation and downregulation of metabolites, respectively. Each group has three biological replicates.

To investigate the functional output of the altered microbiota, we performed untargeted metabolomics on cecal content samples. Orthogonal partial least squares discriminant analysis (OPLS-DA) plots revealed distinct separation patterns in both negative and positive ion modes among the three dietary groups and the control (Figures 2A,B). The analysis indicated significant influences of varying levels of fermented Chinese herbal medicine on the metabolic processes of fattening pigs. Differential metabolites (DMs) identified 124 DMs between the CK and GL (91 negative and 33 positive ions), 33 DMs between CK and GM (21 negative and 12 positive ions), and 26 DMs between CK and GH (13 negative and 13 positive ions). Specifically, the GL exhibited 116 significant metabolite variations (78 up-regulated and 38 down-regulated), the GM showed 25 significant metabolic differences (13 up-regulated and 12 down-regulated), and the GH had 16 notable metabolite changes (10 up-regulated and 6 down-regulated). Further investigation through KEGG pathway analysis explored 19 DMs linked to metabolic pathways involving amino acids, fatty acids, organic acids, nucleotides and proteins (Figure 2C).

### 3.7 Functional interpretation of cecal metabolic shifts via KEGG enrichment

**Figure 3:**
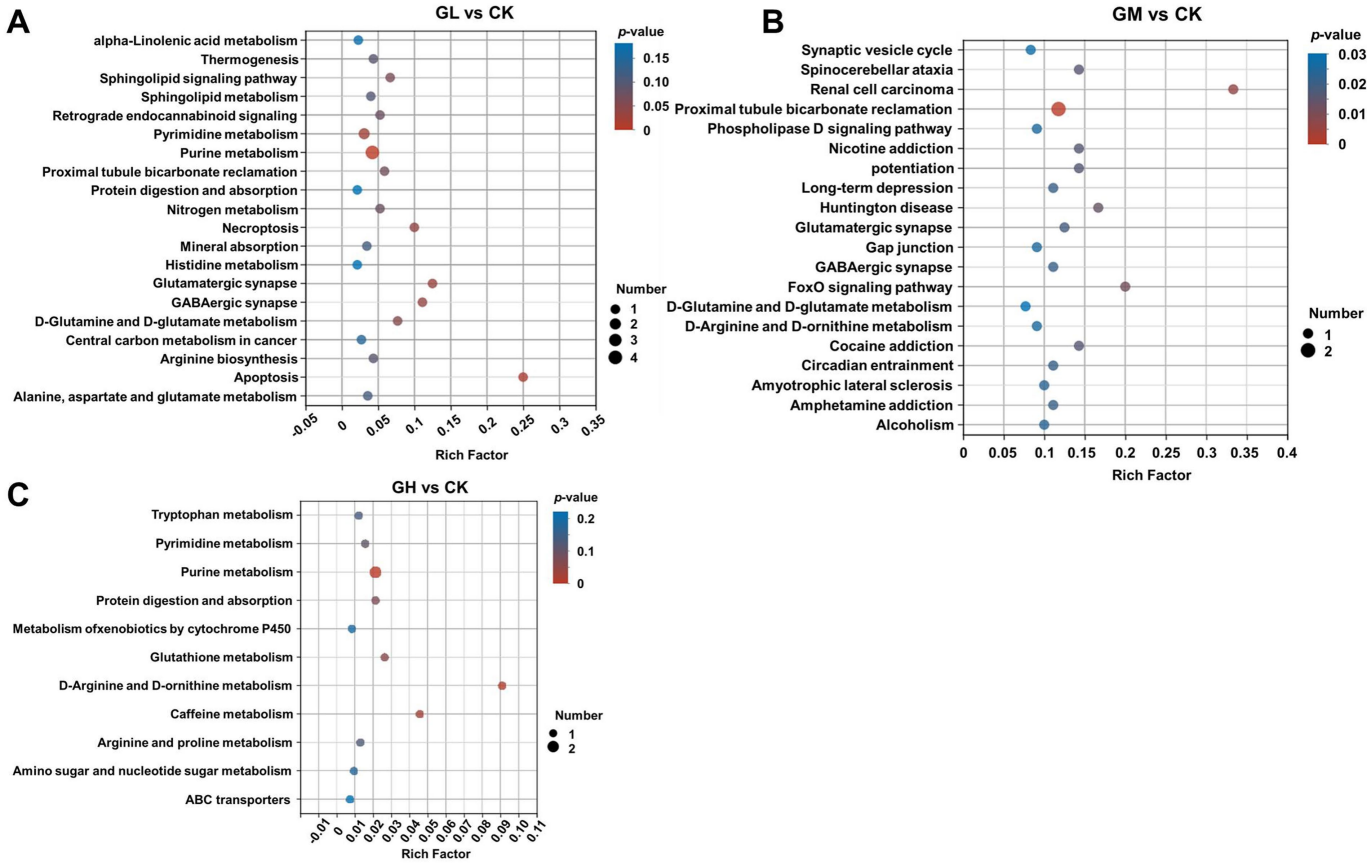
Enrichment analysis of KEGG metabolic pathways associated with various metabolites in the intestine. **(A)** Enrichment of KEGG metabolic pathways of differential metabolites in the gut of GL and CK. **(B)** Enrichment of KEGG metabolic pathways of differential metabolites in the gut of GM group and CK. **(C)** Enrichment of KEGG metabolic pathways of differential metabolites in the gut of GH and CK. The *x*-axis denotes the Rich Factor, while the *y*-axis lists the enriched gene pathway names. Dot size indicates the gene count and dot color signifies the *p*-value magnitude. Each group has three biological replicates.

To elucidate the biological implications of the altered cecal metabolome, we performed Kyoto Encyclopedia of Genes and Genomes (KEGG) pathway enrichment analysis on the identified differential metabolites. In comparison to the CK, the GL showed significant enrichment in metabolic pathways, including purine metabolism, apoptosis, glutamatergic synapse, GABAergic synapse and pyrimidine metabolism. The key metabolites identified in this group were cytosine, L-glutamine, adenosine 3′-monophosphate, guanosine and sphingosine (Figure 3A). The GM primarily exhibited differential metabolites concentrated in D-arginine and D-ornithine metabolism, D-glutamine and D-glutamate metabolism, and glyoxylate and dicarboxylate metabolism pathways. Relevant metabolites included 2′-deoxyinosine, (S)-glutamic acid, L-malic acid, 1,4-diaminobutane, deoxyinosine, deoxyguanosine, uric acid, hypoxanthine and 5-methylcytosine (Figure 3B). Finally, in the GH, differential metabolites were mainly involved in D-arginine and D-ornithine metabolism and purine metabolism, with xanthine, hypoxanthine and 1,4-diaminobutane as the primary metabolites (Figure 3C).

### 3.8 Integrated analysis reveals associations among the cecal microbiota, metabolites, and muscle flavor traits

**Figure 4:**
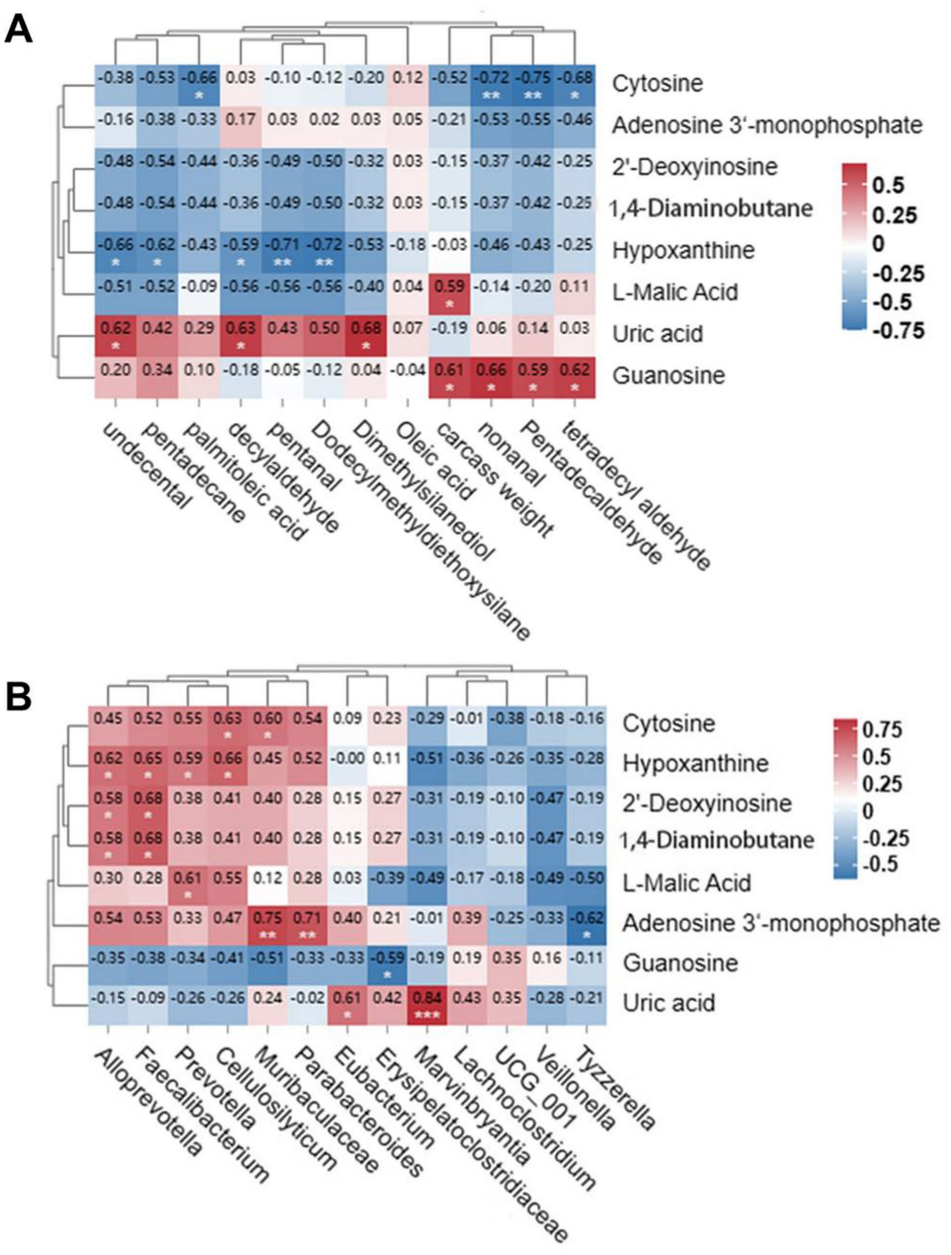
Analysis of the association between intestinal metabolites, meat flavor, and intestinal bacteria genus. **(A)** Heatmap illustrating the relationship between meat flavor parameters and intestinal metabolomic profiles. **(B)** Correlation heatmap depicting the associations between intestinal metabolomic data and cecal bacterial populations. Shades of red and blue denote positive and negative correlations, respectively. Asterisks indicate statistical significance: *p < 0.05 and **p < 0.01.

To explore potential multi-omics links underlying the observed phenotypic changes, we conducted an integrative correlation analysis. This analysis revealed suggestive associations between cecal metabolic profiles and muscle flavor compounds (Figure 4A). Specifically, the gut metabolite guanosine exhibited a positive correlation with 14 different aldehydes, pentadecanal, nonanal and carcass weight. In contrast, uric acid positively correlated with dimethylsilyl diol, decanal and undecenal. Increased carcass weight was associated with elevated levels of L-malic acid, while hypoxanthine showed a negative correlation with several compounds, including 12 alkyl dimethoxy silane, pentanal, decanal, pentadecane, and undecenal. Cytidine demonstrated a negative correlation with 14 aldehydes, pentadecanal, nonanal and palmitic acid. The regulation of purine and pyrimidine nucleotide metabolism significantly influenced the levels of muscle aldehydes and alcohols. Furthermore, metabolic pathways involving organic acids, such as citric acid, acetone acid, acetaldehyde, dicarboxylic acid and bile secretion, had notable effects on alcohols, aldehydes and carcass weight in the longissimus dorsi muscle. Additionally, gut metabolite levels were correlated with bacterial abundance (Figure 4B). Notably, uric acid levels were positively associated with the abundance of *Marvinbryantia* and *Eubacterium*, while 3′-Adenosine monophosphate was negatively linked with *Tyzzerella* and positively with *Parabacteroides* and *Muribaculaceae*. Moreover, xanthine, 2′-deoxyguanosine and 1,4-diaminobutane levels all positively correlated with *Faecalibacterium* and *Alloprevotella* abundance. *Prevotella* and *Cellulosilyticum* abundances were linked to higher xanthine levels, whereas elevated L-malic acid levels correlated positively with *Prevotella* abundance. Interestingly, cytidine levels positively correlated with *Cellulosilyticum* and *Muribaculaceae* abundances, while guanine content was negatively associated with *Erysipelatoclostridiaceae* abundance. Taken together, these exploratory correlations—though derived from a limited sample size and requiring future validation—sketch a preliminary network connecting FCHM-induced modulation of the gut ecosystem to changes in muscle composition and flavor-related traits. It should be noted that this study did not measure blood metabolite profiles, which represent a key intermediate step connecting gut alterations with muscle phenotypes. Therefore, the proposed relationships between gut microbial changes and meat quality traits require further validation through follow-up studies that incorporate systemic circulating metabolite analysis.

## 4 Discussion

With the advancement of modern biotechnology, fermentation technology has been extensively applied and is regarded as an effective method for enhancing the bioactivity of Chinese herbal medicine (Hussain et al., 2016). This technology principally functions by releasing active constituents from herbs and converting plant macromolecules into micromolecules, thereby enhancing nutrient assimilation and lowering the pH of animal feed, consequently improving its taste and nutritional profile (Cai et al., 2012; Li et al., 2020). It is important to clarify that the present study was designed to evaluate the composite fermented Chinese herbal medicine (FCHM) as a whole entity. The fermentation process likely leads to microbial biotransformation, potentially generating new metabolites (e.g., organic acids, enzymes, bacteriocins) and altering the structure and bioavailability of the original herbal components (e.g., polysaccharides, flavonoids). Therefore, the overall effects observed are presumably the result of the combined action of the original herbal constituents and fermentation-derived metabolites. Disentangling their individual contributions is complex and was beyond the scope of this initial application-oriented study. Instead, our work provides an important assessment of the comprehensive application potential of this specific FCHM additive in swine production and establishes a phenotypic and multi-omics framework. This framework, which includes the identified shifts in cecal microbiota and metabolism, will guide future targeted research to identify the specific active compounds responsible for the observed benefits. Current research indicates that incorporating fermented medicinal plants to the feed of fattening pigs can improve growth weight, feed intake and feed conversion ratio (Jeong and Kim, 2015; Cullen et al., 2005; Li et al., 2012). In this experiment, the inclusion of fermented Chinese herbal medicine in the diet of fattening pigs resulted in significantly higher final body weight and average daily gain in the experimental groups, alongside a significant reduction in average daily feed intake and the feed-to-gain ratio, corroborating previous research findings.

The slaughter rate, lean meat percentage, ribeye area and backfat thickness are critical metrics for assessing porcine slaughter performance. Notably, ribeye area and backfat thickness significantly affect the lean meat percentage. Studies have indicated that incorporating unfermented compound Chinese herbal medicine into feeds enhances the slaughter rate and lean meat percentage in fattening pigs while reducing the backfat thickness and fat mass (Du et al., 2012). Ahmed et al. found that including fermented Chinese herbal medicine in pig feed significantly decreased backfat thickness and increased lean meat percentage, without affecting final weight, carcass grade, or ribeye area (Ahmed et al., 2016). In this study, the experimental group treated with fermented Chinese herbal medicine showed a significant increase in slaughter rate and lean meat percentage, and a significant decrease in backfat thickness, whereas the effect on the ribeye area was not significant. Muscle tenderness is a crucial determinant of meat palatability, with increased tenderness generally improving eating quality. Additionally, the water retention capacity of muscles is closely related to their pH value (Schafer et al., 2002). Upon adding fermented Chinese herbal medicine to the feed, this trial observed a downward trend in shear force, with cooking loss and muscle pH values remaining within normal limits.

In this experiment, oleic acid was the most abundant fatty acid, followed by palmitic and stearic acids, consistent with Ahmed’s findings (Ahmed et al., 2016). Related studies indicate that the tastiness of meat products is linked to both the phospholipid level and their unsaturation. The content of unsaturated fatty acids (UFAs), mainly oleic and linoleic acids, is a critical factor for assessing meat palatability. Oleic acid, known for its cholesterol-lowering effect and often referred to as a “safe fatty acid,” contributes to the flavor profile of meats (Xiang et al., 2018). A higher UFA content signifies superior meat flavor (Hoa et al., 2020). In this study, the addition of traditional Chinese herbal medicine tended to increase the content of polyunsaturated fats, particularly in the GM, suggesting an enhancement in pork’s taste and aroma profile. The oxidation of lipid and minor fatty acid play a crucial role in modulating meat flavor. Specific fatty acids, such as oleic acid (C18:1), elaidic acid (C18:1), heptadecenoic acid (C17:1), linoleic acid (C18:2), stearic acid (C18:0) and palmitoleic acid (C16:1), have been identified as potential flavor precursors in pork metabolism (Song et al., 2017). This study found that dietary inclusion of fermented Chinese herbal medicine significantly increased the levels of oleic, elaidic and palmitoleic acids in muscle fatty acids, contrasting with the CK group, while stearic acid levels remained relatively unchanged, indicating an accumulation of flavor precursor metabolites following herbal supplementation. High saturated fatty acid (SFA) content is linked to increased cholesterol and a heightened risk of cardiovascular and coronary heart diseases. Increased levels of SFAs such as lauric acid (C12:0), myristic acid (C14:0) and palmitic acid (C16:0) are detrimental to human health (Sha, 2009). In this study, lauric acid content in pork was significantly reduced with fermented Chinese herbal medicine, and the overall SFA content did not change significantly, suggesting that the addition of Chinese herbal medicine does not affect pork’s antioxidant capacity.

Volatile flavor compounds are largely generated through lipid oxidation. Aldehydes, as major products of this process, play a central role in the formation of meaty aromas (Xie et al., 2008; Rasinska et al., 2019). These aldehydes impart distinct aromatic profiles to pork; for instance, pentanal evokes malt and roasted nut aromas, nonanal exudes scents of fat and citrus, while decanal possesses rubbery and oily olfactory notes. A higher concentration of nonanal is typically associated with a fresher meaty scent (Nieto et al., 2011). This study found that the addition of Chinese herbal medicines led to an increase the content of nonanal and benzaldehyde in pork. Furthermore, the production of alcohols, primarily derived from the decomposition of conjugated linoleic acid, enhances meat flavor formation (Ma et al., 2012).

The study revealed an increase in the content of dimethicone diol across all experimental groups. Wang et al. (2017) have indicated that the addition of probiotics significantly enhances the variety of flavor compounds in meat. In this trial, the diversity of flavor substances in the experimental group surpassed that of the CK group, aligning with the findings of Wang et al. Esters, formed through the interaction of free fatty acids and oxidized alcohols, are responsible for the fruity aromas in pork. Notably, the ester content in all experimental groups was significantly higher than in the CK group, with the highest levels observed in the GM group. Collectively, these findings suggest that Chinese herbal medicines may enhance pork flavor and expand its aromatic profile by modulating lipid oxidation and flavor precursor metabolism.

The equilibrium of the gut microbiota is crucial for the development of the animal’s intestinal system and its overall health. The microbiota primarily facilitates the digestion and absorption of dietary nutrients, enhance host metabolic processes, and optimize nutrient utilization efficiency. The diversity and dynamics of the gut microbiome are influenced by factors such as age, health status, dietary patterns, breed and environmental conditions, with diet exerting the most rapid and notable effects (Garre and Modrego, 2024; Palmer et al., 2007). Our study, utilizing 16S rRNA sequencing analysis of cecal samples, found that varying levels of fermented herbal medicine can significantly alter the structure of the gut microbial community. At the phylum level, as the amount of fermented Chinese herbal medicine added gradually increased, an upward trend in the abundance of Firmicutes was observed. Notably, Firmicutes are prolific producers of butyrate, a compound that is instrumental in regulating energy metabolism and elevating leptin gene expression (Fei and Zhao, 2013). These results suggest that the incorporation of fermented Chinese herbal medicine may increase lean meat percentage and diminish backfat thickness.

At the genus level, the relative abundance of *Muribaculaceae* was significantly reduced in the experimental group. *Muribaculaceae* are important polysaccharide-degrading bacteria in the gut, often considered beneficial commensals involved in dietary fiber fermentation (Liu, 2021; Liu et al., 2021). Their reduced abundance may be related to FCHM altering the type or availability of fermentable carbohydrates in the gut. Studies have shown that high-fat diets can reduce *Muribaculaceae* levels in murine gut microbiota (Zhao et al., 2021). In this study, although the content of *Muribaculaceae* was significantly reduced, the growth performance of the experimental group was unaffected. However, whether a diet-induced change in a specific bacterial taxon is beneficial must be judged in the context of overall host physiology. In this study, despite the decrease in *Muribaculaceae* abundance, the growth performance of the experimental pigs was not adversely affected, and some beneficial metabolic shifts were observed. Therefore, the change in *Muribaculaceae* induced by FCHM is more likely part of a holistic reshaping of the gut microbial ecosystem, and its specific functional implications require interpretation with additional functional genomic data. Furthermore, the abundance of *Faecalibaculum* showed a positive correlation with blood lipid levels (Han et al., 2024) and was significantly reduced in this experiment, indicating that fermented Chinese herbal medicine additions might help mitigate host blood lipid concentrations.

The accumulation of nucleotides and amino acids in the cecum may suggest altered intestinal microbial metabolic activity or enhanced processing of dietary components (Liu et al., 2021). Research in other species indicates that microbial metabolic products of nucleotides and related compounds can influence meat quality and flavor profiles. Our untargeted metabolomics revealed that FCHM supplementation altered the cecal abundance of various metabolites. Notably, a significant reduction in the level of hypoxanthine—a central intermediate in the microbial purine degradation pathway—was observed in the cecal content of the GM and GH groups. Certain bacteria are known to rapidly convert nucleotides like inosine into hypoxanthine (Hong et al., 2017), which is further metabolized to compounds such as xanthine and uric acid (Jääskeläinen et al., 2019). However, it is important to recognize the distinct physiological contexts of this metabolite. In muscle, hypoxanthine primarily accumulates post-mortem as an end-product of ATP degradation and is a recognized indicator of meat freshness and flavor development in that specific tissue. In the gut, it is a transient intermediate of microbial purine metabolism. Therefore, the reduction in cecal hypoxanthine should not be interpreted as a direct contributor to muscle flavor. Rather, this finding provides evidence that FCHM supplementation altered the dynamics of microbial nitrogen metabolism in the large intestine. This modulation of gut microbial metabolism may have secondary, systemic consequences on the host’s nitrogen economy and energy metabolism, which could, in turn, create a physiological milieu conducive to the observed improvements in muscle composition (e.g., fatty acid profile). This reframed interpretation separates the site-specific observation from the distal outcome while maintaining a plausible, if indirect, connection via host metabolism. It is noteworthy that excessive systemic accumulation of purine degradation products like uric acid is associated with adverse health outcomes (Zheng et al., 2018). Therefore, the observed decrease in cecal hypoxanthine suggests that FCHM may modulate the microbial purine metabolism pathway within the gut. This shift could influence the intestinal pool of flavor-precursor metabolites and their downstream products, potentially affecting the substrates available for absorption and subsequent deposition in muscle tissue, thereby contributing to the observed modifications in muscle composition.

**Figure 5:**
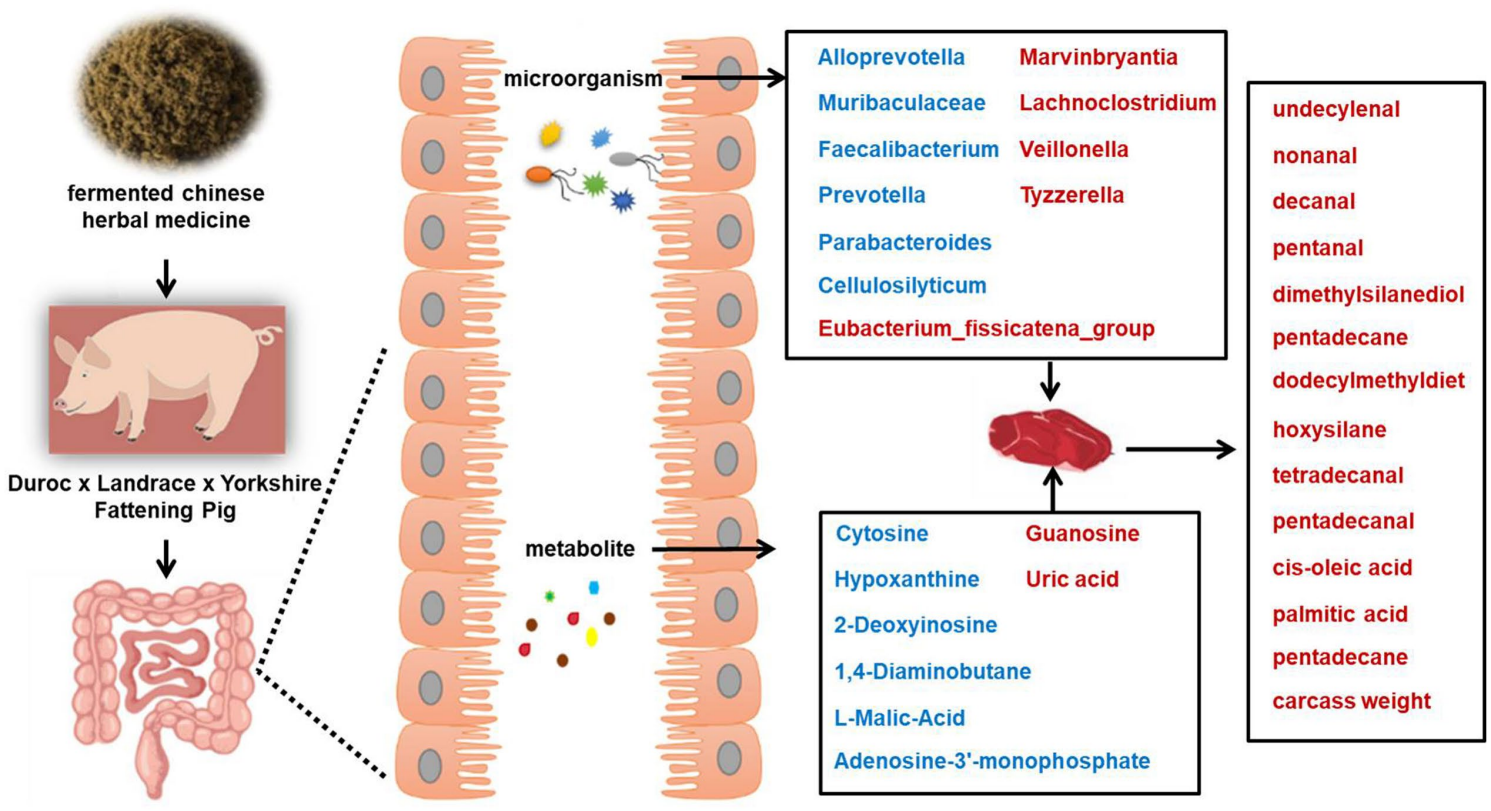
Schematic diagram illustrating the putative mode of action and correlations between gut microbial communities, cecal metabolomics, and meat flavor changes in fattening pigs fed fermented Chinese herbal medicine (FCHM). The diagram depicts the proposed chain: Dietary FCHM supplementation reshapes the cecal microbiota and metabolome, which are associated with improved growth performance and favorable changes in muscle fatty acids and volatile flavor compounds. Metabolites, bacteria, and muscle compounds that were significantly downregulated are denoted in blue, while those upregulated are indicated in red.

This study demonstrated a significant enrichment of the tricarboxylic acid (TCA) cycle and related dicarboxylic acid metabolism in the GM group. As a central hub for energy metabolism, the TCA cycle facilitates the complete oxidation of substrates derived from carbohydrates, fats, and proteins, thereby promoting efficient energy harvesting and utilization (Arnold and Finley, 2023). The enrichment of this pathway, coupled with the specific microbial and metabolic shifts observed, suggests potential associations between gut microbial changes and muscle phenotypes, offering a working hypothesis for how FCHM may influence host physiology. Based on the multi-level changes observed in this study, we propose a putative mode of action for the FCHM additive: (1) As a microbiome modulator, the composite fermented product provides bioactive compounds and fermentation metabolites that selectively reshape the cecal microbial community structure (e.g., increasing *Marvinbryantia* and *Veillonella*, decreasing *Alloprevotella* and *Prevotella*). (2) The restructured microbiota alters its functional output, particularly influencing microbial metabolic pathways in the cecum, such as purine metabolism (evidenced by changes in hypoxanthine and uric acid) and energy metabolism (e.g., TCA cycle intermediate L-malic acid). (3) These local changes in the gut ecosystem, possibly through the absorption of microbial metabolites (e.g., SCFAs) or via systemic metabolic adjustments, ultimately contribute to improved host growth performance (increased ADG, reduced F/G), optimized muscle fatty acid profile (e.g., elevated oleic acid), and an enriched spectrum of volatile flavor compounds (e.g., increased nonanal, decanal). This integrative view of microbiota, metabolism, and host phenotype offers a mechanistic framework for understanding the multifaceted benefits of FCHM supplementation. As summarized in Figure 5, the supplementation led to a restructuring of the cecal microbiota (e.g., downregulation of *Alloprevotella* and *Muribaculaceae*; upregulation of *Marvinbryantia* and *Veillonella*) and consequent changes in the microbial metabolome (e.g., alterations in nucleotides like hypoxanthine and key intermediates like L-malic acid). These gut-level changes are correlated with the improved muscle composition, including an optimized fatty acid profile (e.g., increased cis-oleic acid) and a refined spectrum of volatile flavor compounds (e.g., increased nonanal, decanal). Collectively, our findings outline a potential multi-step mechanistic hypothesis: FCHM remodels the gut ecosystem, which modulates host-microbial co-metabolism, and these changes are associated with an altered biochemical profile in muscle tissue. This working model provides a framework for future targeted investigations. It is crucial to note that this study did not profile serum metabolites, which represent the critical intermediary compartment connecting gut content changes to peripheral tissue phenotypes. Therefore, the proposed “gut-muscle” axis remains a hypothesis that requires direct validation through future studies incorporating serial blood metabolomic analyses to trace metabolite flux and establish causal links.

This study possesses several notable strengths, including the first integrated multi-omics characterization of FCHM effects in finishing pigs, encompassing growth performance, carcass traits, meat quality, gut microbiota, and metabolomic profiles. The comprehensive phenotypic assessment from a full cohort of 76 pigs, combined with exploratory multi-omics integration, establishes a valuable framework linking dietary intervention, gut microbial ecology, and muscle composition.

Nevertheless, certain limitations warrant consideration. The microbiome and metabolome analyses were performed on a limited subset of animals (*n* = 3 per group). While this subset was carefully selected based on body weight to minimize variation, the small sample size limits statistical power and generalizability; therefore, the reported microbial and metabolic shifts must be considered preliminary and hypothesis-generating rather than definitive conclusions. Additionally, the absence of serum metabolomic data constitutes a critical gap in our proposed framework. Without profiling blood metabolites, we cannot trace the specific metabolic flux from the intestinal lumen to peripheral tissues such as skeletal muscle, nor confirm which metabolites serve as direct mediators of the observed muscle changes. Consequently, the causal pathways linking gut microbial alterations to meat quality traits remain hypothetical rather than established.

Despite these limitations, this work provides a valuable multi-dimensional dataset that establishes FCHM supplementation as a promising strategy for improving growth performance and meat quality in finishing pigs. More importantly, it generates a clear set of testable hypotheses and identifies specific biomarkers and pathways—including purine metabolism intermediates, tricarboxylic acid cycle metabolites, and key bacterial genera—that warrant targeted investigation in future studies. To move beyond correlation toward causation, future research should prioritize large-scale validation cohorts, serial blood metabolomic profiling, and comparative chemical characterization of fermented products. Addressing these priorities will be essential to fully realize the potential of fermented herbal products as sustainable strategies for improving animal production and meat quality.

## 5 Conclusion

This study indicates that dietary supplementation with 0.4–0.8% fermented Chinese herbal medicine in finishing pigs is associated with enhanced growth performance and carcass quality, alongside favorable shifts in muscle composition related to flavor. The 0.6% supplementation level suggested the most integrated benefits. These effects may be mediated through a modulation of the cecal microbiota, including the enrichment of genera such as *Marvinbryantia*, *Veillonella*, and *Tyzzerella* and the suppression of *Alloprevotella*, *Muribaculaceae*, and *Prevotella*. Concomitant changes in the cecal metabolome involved key pathways such as purine metabolism and the tricarboxylic acid cycle, reflected by elevated levels of beneficial metabolites including L-malic acid, uric acid, and guanosine, and a reduction in hypoxanthine. The multi-level alterations observed in the gut ecosystem corresponded to an optimized muscle fatty acid profile, characterized by an increased proportion of unsaturated fatty acids such as oleic acid, and a refined spectrum of volatile flavor compounds marked by the enrichment of characteristic aldehydes. This work provides preliminary mechanistic insights into how FCHM influences pork quality through modulation of the gut microbiota and their metabolites. The observed multi-level associations establish a comprehensive framework linking dietary intervention, gut microbial ecology, metabolic pathways, and muscle composition. These findings offer a valuable foundation for future research to further elucidate the mechanisms underlying the beneficial effects of fermented herbal products in sustainable animal production.

## Data Availability

The datasets presented in this study are available in online repositories and can be found with the accession number accession number SRP682478. (https://www.ncbi.nlm.nih.gov/sra/SRP682478).
