# Supplementary material for: Exploring the effects of fermented Chinese herbal medicine on growth, cecal microbiota, metabolism, and muscle flavor-related compounds in fattening pigs
Source: Front Microbiol. 2026 Mar 25;17:1781152. doi: 10.3389/fmicb.2026.1781152 (PMC13056876; doi:10.3389/fmicb.2026.1781152)
Supplement: Supplementary file 1 [file Table_1.docx]

**Supplementary materials**

**Table S1 The content of Chinese herbal medicine**

| **Composition** | **Content (%)** | **Composition** | **Content (%)** |
| --- | --- | --- | --- |
| Tangerine peel | 13.33 | Cloves | 6.67 |
| Fennel | 13.33 | Nutmeg | 6.67 |
| Staranise | 13.33 | Ginger | 6.67 |
| Cinnamon | 13.33 | Hawthorn | 6.67 |
| Wolfberry | 13.33 | Garlic powder | 6.67 |
