# Supplementary material for: Exploring the effects of fermented Chinese herbal medicine on growth, cecal microbiota, metabolism, and muscle flavor-related compounds in fattening pigs
Source: Front Microbiol. 2026 Mar 25;17:1781152. doi: 10.3389/fmicb.2026.1781152 (PMC13056876; doi:10.3389/fmicb.2026.1781152)
Supplement: Supplementary file 2 [file Table_2.docx]

**Supplementary materials**

**Table S2 The information of fermentation strains**

| Strains type | Strain name | Isolate number |
| --- | --- | --- |
| Saccharomycetes | *Pichia yeast* JZ10 | CCTCC M 2020843 |
|  | *Saccharomyces cerevisiae* JZ9 | CCTCC M 2020844 |
| *Lactobacillus* | *Lactobacillus casei* YLW003 | CCTCC M 2018761 |
|  | *Lactobacillus rhamnosus* YLW001 | CCTCC M 2018759 |
|  | *Pediococcus acidilactici* YLW002 | CCTCC M 2018760 |
