# Supplementary material for: Exploring the effects of fermented Chinese herbal medicine on growth, cecal microbiota, metabolism, and muscle flavor-related compounds in fattening pigs
Source: Front Microbiol. 2026 Mar 25;17:1781152. doi: 10.3389/fmicb.2026.1781152 (PMC13056876; doi:10.3389/fmicb.2026.1781152)
Supplement: Supplementary file 3 [file Table_3.docx]

**Supplementary materials**

| **Table S3 The nutritional components, bioactive substances, and toxin substances in feed before and after fermentation** | | |
| --- | --- | --- |
| Items | Before fermentation | After fermentation |
| **Nutritional composition** |  |  |
| Soluble sugar (SS), mg/g | 27.57 ± 3.25^b^ | 41.33 ±1.19^a^ |
| Neutral detergent fiber (NDF), % | 42.90 ± 0.76 | 41.48 ± 0.28 |
| Acid Detergent Fiber (ADF),% | 36.70 ± 0.71 | 34.68 ± 0.61 |
| Lactic acid (LA), mg/g | 1353.43 ± 41.41 | 1433.71 ± 55.65 |
| Acetic acid (AA), mg/g | 3.45 ± 0.44^b^ | 4.96 ± 0.44^a^ |
| pH | 4.23 ± 0.023^a^ | 4.11 ± 0.023^b^ |
| **Bioactive substances** |  |  |
| Total polysaccharides (TPs), mg/g | 9.46 ± 1.19^a^ | 13.94 ± 1.43^b^ |
| Alkaloid (AKD）, mg/g | 0.036 ± 0.0040^b^ | 0.16 ± 0.013^a^ |
| Flavonoid (FVD), mg/g | 23.00 ± 0.61 | 21.59 ± 0.35 |
| Total saponins (TSs), mg/g | 54.10 ± 2.45^b^ | 64.62 ± 1.71^a^ |
| **Toxic substances** |  |  |
| Deoxynivalenol (DON), μg/kg | 632.73 ± 14.47a | 526.54 ± 12.45^b^ |
| Aflatoxin (AFB1), μg/kg | 33.33 ± 0.53^a^ | 31.29 ± 0.71^b^ |
| Zearalenone (ZEN), μg/kg | 18.65 ±0.43^a^ | 16.65± 0.63^b^ |
