# Supplementary material for: Exploring the effects of fermented Chinese herbal medicine on growth, cecal microbiota, metabolism, and muscle flavor-related compounds in fattening pigs
Source: Front Microbiol. 2026 Mar 25;17:1781152. doi: 10.3389/fmicb.2026.1781152 (PMC13056876; doi:10.3389/fmicb.2026.1781152)
Supplement: Supplementary file 4 [file Table_4.docx]

**Supplementary materials**

**Table S4. Feed formula and nutritional level**

| Raw material | Content（%） | Nutrient levels (%) |  |
| --- | --- | --- | --- |
| Corn | 57.10 | DE/(MJ/Kg) | 11.80 |
| Wheat bran | 15.60 | CP | 14.00 |
| Brewery mash | 10.40 | Lys | 0.51 |
| Rapeseed cake | 7.70 | Cys | 0.53 |
| Soybean meal | 5.20 | Thr | 0.50 |
| Premix^1^ | 4.00 | Try | 0.15 |
| Total | 100.00 | Ile | 0.50 |
|  |  | Ca | 0.70 |
|  |  | TP | 0.50 |
|  |  | NPP | 0.16 |
|  |  | Na | 0.05 |
|  |  | Cl | 0.05 |

The premix provided the following per kilogram of diets: VA 6000 IU, VD 2000 IU, VE 30.00 mg, VK 31.50 mg, VB_1_ 12.00 mg, VB_4_ 25.00 mg, VB_6_ 62.50 mg, VB_12_ 0.20 mg, nicotinic acid 20.00 mg. Calcium pantothenate 13.00 mg, folic acid 0.35 mg, biotin 0.30 mg, choline 300 mg, Fe 80 mg, Cu 15 mg, Zn 80 mg, Mn 20 mg, I 0.50 mg, Se 0.25 mg.
